# Extract from a Letter from Alphonso Severance, M. D., to the Senior Editor, with Wood Cuts and Description

**Published:** 1858-07

**Authors:** 


### ARTICLE VI.

*Extract from a Letter from* ALPHONSO SEVERANCE, M. D., *to the Senior Editor, with wood cuts and description.*

DEAR SIR:

I wish to communicate to you my method of taking wax impressions. I think it especially applicable in cases where there is a very deep alveolar border, and I prefer the wax holder that I am about to describe in *all* cases, as it is as convenient to use as any other. It always insures a perfect impression of the highest point of the roof of the mouth. With the common wax holder I have always had a difficulty in doing this where the arch is very deep.

In your "Principles and Practice" you describe one made with a hole through it, that the operator may push the wax further up, merely with the fingers. Mine is on the same principle, but with the addition of what I believe to be quite an improvement. It consists of two separate pieces, the larger one made the same as you describe, with the exception of the opening being much smaller, merely large enough for a small point on the lower side of the smaller piece, to pass through.

The small part is made to fit and to cover the central portion of the large one, with the before mentioned point of the small one to pass through.

This is made long enough to be pushed up close to the roof of the mouth, after the large one is forced up as high as desirable, while taking the impression.

I have drawn the different parts sufficiently plain to give you a distinct idea of the apparatus. I am not able with the "pencil," to give the exact form of the larger piece, but I will try to do so with the "pen."

I make it of the form of an atmospheric full plate, where the mouth is quite flat, the small piece enabling the operator to force up the wax to any required distance.

I invented this article several years ago, and have exclusively made use of it ever since, and think I could not hardly get along with any other. By means of it, I am enabled always to get a perfect fit to my plates, in the highest part of the roof of the mouth, and I cannot make sure of this with any other instrument. Besides, it is done with no more trouble than with the ordinary wax holder, as you will readily perceive.

I prefer to make them of either pure block tin, or of some similar metal that can be readily cast, instead of striking them up with dies. I made my first or original pattern from plaster of paris, moulding it in sand with a *separating flask*, and casting the metal into the sand. Should I wish to manufacture many of them, I should have a brass or iron mould made to cast them into.

FIG. 1.

FIG. 2.

FIG. 3.

*a*

*b*

*c*

Fig. 1. Wax holder, *upper view, without* the smaller portion.

Fig. 2. The same, *with smaller piece on.*

Fig. 3. Lateral view, with small part attached.

*a*, upper view of small portion, convex.

*b*, under view, concave, with small point or projection to pass through aperture in Fig. 1.

*c*, end view of small piece, upper part up.

GREAT FALLS, N. H., *March* 24, 1858.

## Figures and Tables

**Fig. 1. f1:**
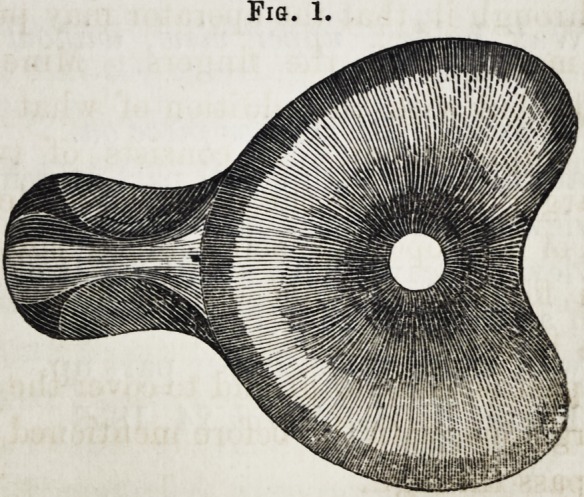
Wax holder, *upper view, without* the smaller portion.

**Fig. 2. f2:**
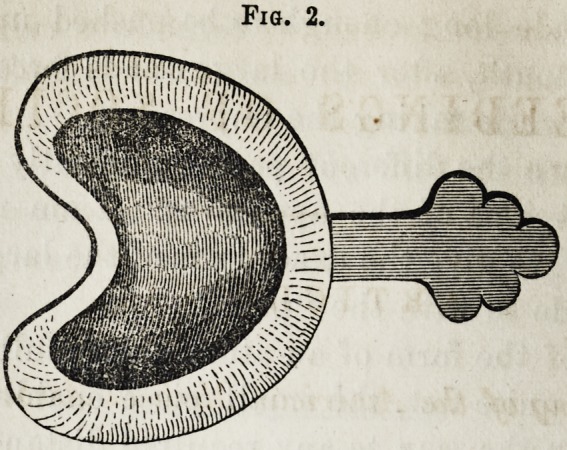
The same, *with smaller piece on.*

**Fig. 3. f3:**
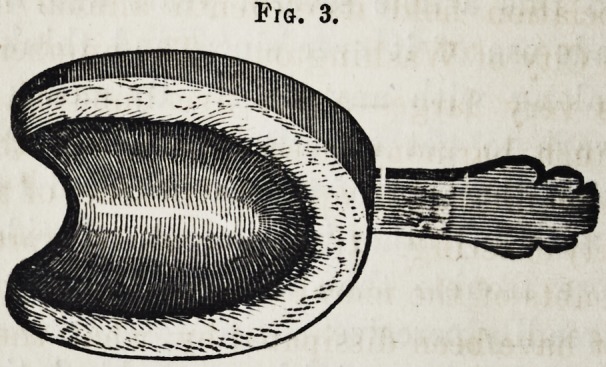
Lateral view, with small part attached.

**a f4:**
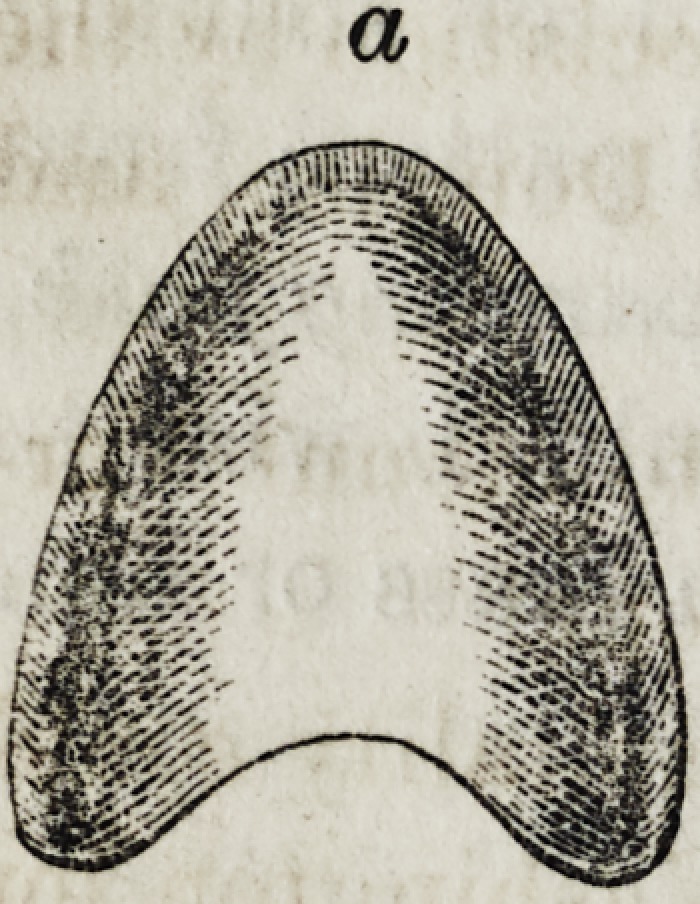
upper view of small portion, convex.

**b f5:**
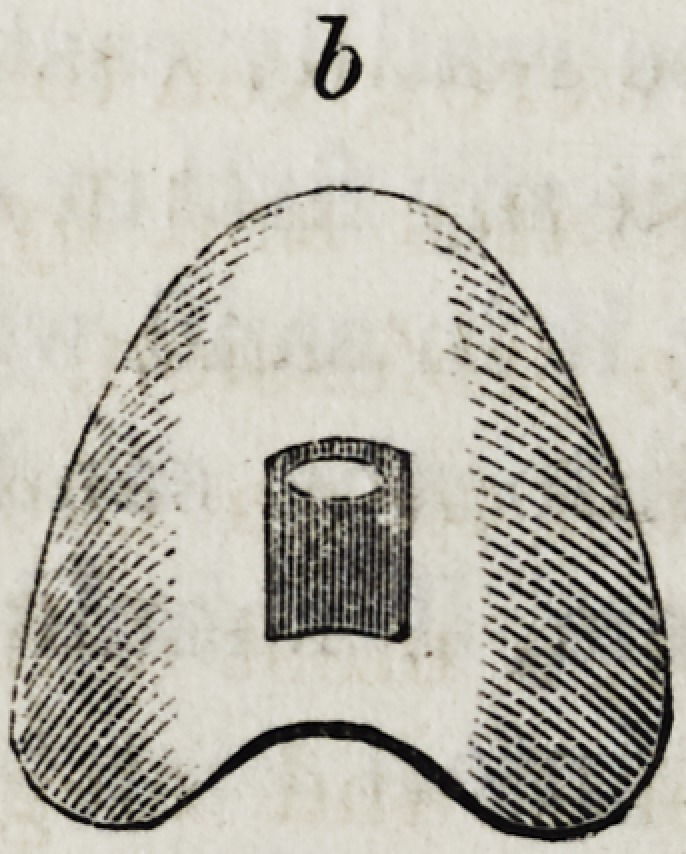
under view, concave, with small point or projection to pass through aperture in Fig. 1.

**c f6:**
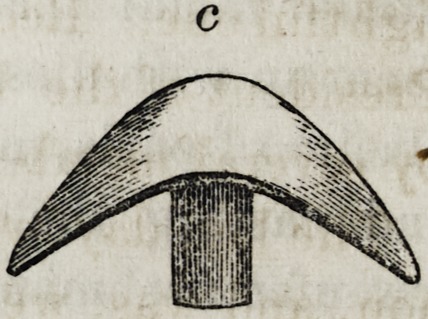
end view of small piece, upper part up.

